# Factors that determine variation in ambulance utilisation

**DOI:** 10.29045/14784726.2019.03.3.4.46

**Published:** 2019-03-01

**Authors:** John Martin

**Affiliations:** College of Paramedics

## Abstract

**Aims:**

This study explored the behaviour of populations in utilising the ambulance service. The research question was ‘What are the factors that determine variation in ambulance utilisation?’ and the aim was to develop further the model of the behaviour.

The following objectives were used with three acuity levels of call:

- To what extent do socio-demographic factors account for variation in ambulance utilisation?
- To what extent does the general health of a population moderate ambulance utilisation?
- To what extent does self efficacy moderate ambulance utilisation?
- To what extent do social and support networks moderate ambulance utilisation?
- To what extent does access to services moderate ambulance utilisation?

**Methods:**

The study analysed 769,376 emergency calls from one ambulance service over a year, including both conveyed and non-conveyed; adjustments were made for workday population shifts. The ambulance calls were sorted into three acuity groups. The calls were matched to socio-demographic factors of the population collected in the census at lower super output area (LSOA). Factors explored included age, gender, socio-economic status (SES), country of birth and ethnicity. Population datasets were utilised to test the moderating effect of self-efficacy, social and support networks, general health status and access to services on the socio-demographic factors.

The study utilised correlation analysis, geographical mapping and moderated multiple-regression analysis. This is a big-data study utilising the principle of ecological correlation.

**Results:**

An overall significant regression was found (F(27,3582) = 44.81, p < .001), with adjusted R^2^ of 0.2469. Population factors related to higher utilisation included:

- Age 85–90 (0.067)
- Age 90 and over (0.114)
- Ethnicity mixed (0.082)
- Ethnicity black (0.071)
- European country of birth (0.044)
- SES group 8 (0.278)

Social and support networks and self-efficacy were not significant moderators of ambulance utilisation. However, access to services (ΔR^2^ = 0.0082) and general health status (ΔR^2^ = 0.0309) were both significant moderators (p < 0.001).

**Conclusion:**

The study concluded that the socio-demographics of a population account for 25% of variation in utilisation. This was moderated by general health status and access to services. However, self-efficacy and social and support networks were not shown to moderate the health behaviour.

Implications for practice include considering policy interventions targeting specific population types related to higher utilisation to manage demand, as well as development of the theoretical model of ambulance utilisation behaviour.

